# The Role of Grain Boundary Diffusion in the Solute Drag Effect

**DOI:** 10.3390/nano11092348

**Published:** 2021-09-10

**Authors:** R. K. Koju, Y. Mishin

**Affiliations:** Department of Physics and Astronomy, George Mason University, MSN 3F3, Fairfax, VA 22030, USA; rkoju@gmu.edu

**Keywords:** atomistic simulation, grain boundary migration, grain boundary diffusion, solute drag

## Abstract

Molecular dynamics (MD) simulations are applied to study solute drag by curvature-driven grain boundaries (GBs) in Cu–Ag solid solution. Although lattice diffusion is frozen on the MD timescale, the GB significantly accelerates the solute diffusion and alters the state of short-range order in lattice regions swept by its motion. The accelerated diffusion produces a nonuniform redistribution of the solute atoms in the form of GB clusters enhancing the solute drag by the Zener pinning mechanism. This finding points to an important role of lateral GB diffusion in the solute drag effect. A 1.5 at.%Ag alloying reduces the GB free energy by 10–20% while reducing the GB mobility coefficients by more than an order of magnitude. Given the greater impact of alloying on the GB mobility than on the capillary driving force, kinetic stabilization of nanomaterials against grain growth is likely to be more effective than thermodynamic stabilization aiming to reduce the GB free energy.

## 1. Introduction

Metallic materials with grain sizes on the order of nanometers have attracted significant technological interest due to many superior properties that they often demonstrate over the traditional coarse-grained materials [1]. The beneficial properties of nanocrystalline materials originate from the large specific area (per unit volume) of the grain boundaries (GBs), phase boundaries, and other internal interfaces [2,3]. In particular, the high mechanical strength of nanocrystalline materials is largely due to the restraining effect imposed by the GBs on the dislocation glide. The most common strengthening mechanism in polycrystalline materials is the formation of dislocation pileups stopped at the GBs [4]. This mechanism, known as the Hall–Petch effect [4], operates at grain sizes down to about 20 nm. At even smaller grain sizes, the mechanical strength plateaus and then reverses as the dislocation sources inside the grains cease to operate and the dislocation pileups can no longer form. Instead, plastic deformation becomes predominantly controlled by GB processes such as GB sliding and grain rotation [5,6,7,8,9,10,11,12,13,14]. The exact mechanisms of the GB-mediated plasticity depend not only on the grain size but also the structure and chemical composition of the GBs. Chemical effects, such GB segregation and GB chemical reactions, strongly impact the sliding resistance of GBs and their mobility in alloy systems.

Broader technological applications of nanocrystalline alloys are hampered by the grain growth at elevated temperatures and in some cases even at room temperature. The grain growth causes degradation of the mechanical strength and the loss of other superior properties of nanocrystalline materials. Thermodynamically, the grain growth is driven by the system’s “desire” to reduce the total GB free energy. Given that the specific GB area in nanocrystalline materials is large, the capillary force driving the grain growth is exceedingly strong.

Over the past decades, significant research efforts have been devoted to finding ways to prevent, or at least reduce, the grain growth in nanomaterials. Most of the research has been focused on alloying the material with suitable solutes. There are two possible approaches to stabilizing nanograins by alloying. The thermodynamic approach is to stabilize the grain structure by reducing the GB free energy γ, and thus the capillary driving force, due to solute segregation to GBs [15,16,17,18,19,20,21,22,23,24,25,26,27,28]. The kinetic approach seeks to stabilize the grains by reducing the GB mobility. The latter goal can be achieved by either causing the solute drag effect [28,29,30,31] or by Zener pinning of GBs by small precipitates of a second phase [28,31,32,33,34,35,36,37]. The Zener pinning mechanism can only be implemented in alloys where the solute elements have an extremely low solubility in the matrix and precipitate in the form of highly disperse nanoparticles resistant to coarsening at the relevant temperatures. This requirement imposes a stringent constraint on the system selection. However, when the required disperse microstructure can be created, the Zener mechanism provides the strongest kinetic stabilization of the nanomaterial.

A recent example of a kinetically stabilized nanomaterial is offered by the immiscible Cu–Ta alloys [37,38,39,40,41,42,43,44]. The face centered cubic (FCC) Cu and body centered cubic (BCC) Ta are practically immiscible in the solid state. High-energy mechanical alloying produces a thermodynamically unstable random solid solution of the two elements. During the subsequent thermal processing, Ta atoms precipitate from the solution in the form of nanometer-scale clusters coherent with the Cu matrix. These clusters strongly pin Cu GBs by the Zener mechanism, preventing grain growth at high temperatures up to the melting point of Cu [44,45,46]. In addition to blocking the dislocation transmission across the GBs, the clusters residing at GBs also suppress the operation of GB-mediated deformation mechanisms (sliding and grain rotation). This double-strengthening leads to unique mechanical properties of Cu–Ta alloys, such as a high strength (above 1 GPa), small strain-rate sensitivity [40], and excellent creep resistance [47].

Kinetic stabilization by solute drag is not as effective as the Zener pinning, but this approach is more general and applicable to a much broader spectrum of nanomaterials. The stabilization process can be controlled by optimizing the chemical composition, texture, and thermal processing, which requires a fundamental knowledge of the solute–GB interactions and their impact on the strength of the solute drag.

**Figure 1:**
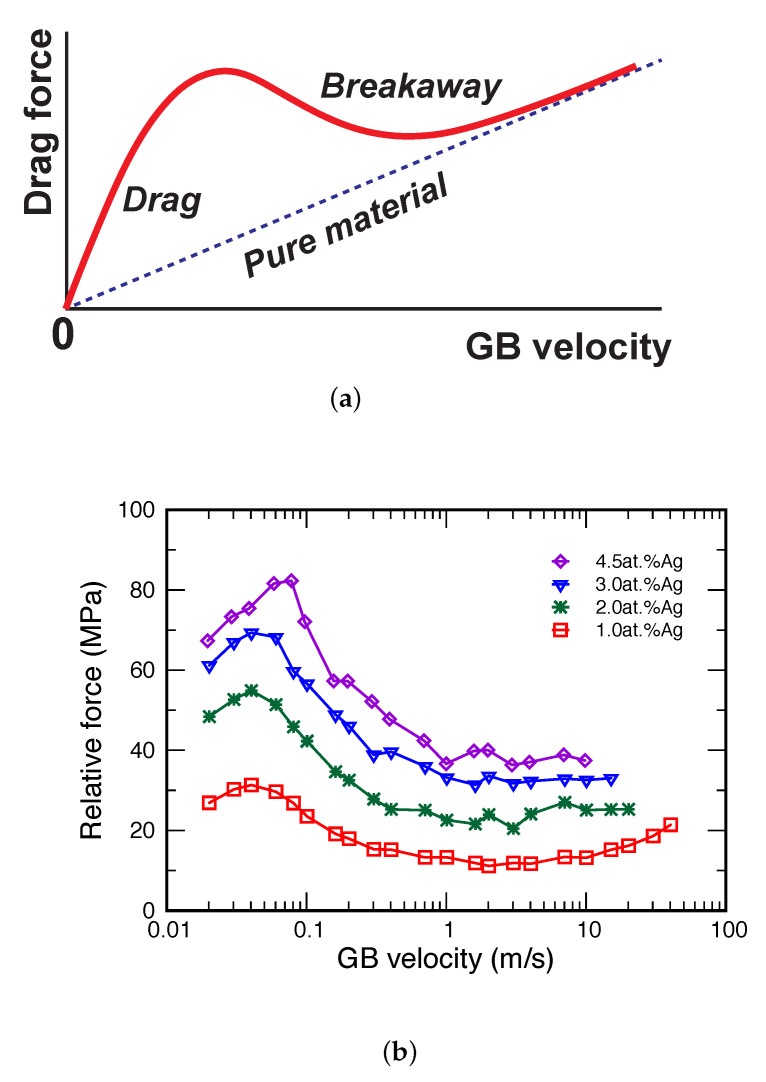
(**a**) Schematic force–velocity diagram according to the classical model of GB solute drag [29,48,49]. The maximum of the drag force separates two kinetic regimes, with the GB dragging the segregation atmosphere at low velocities and breaking away from it at high velocities. (**b**) Driving force for the planar Σ17(530)[001] GB as a function of GB velocity at different alloy compositions is indicated in the key [50]. The GB is driven by an applied shear stress.

The solute drag by GBs has been the subject of numerous experimental, theoretical, and modeling studies over several decades. The classical model proposed by Cahn [29] and Lücke et al. [48,49] predicts a highly nonlinear relation between the GB velocity and the drag (friction) force, with a maximum of the drag force reached at some critical velocity (Figure 1a). On the low-velocity side of the maximum, the segregation atmosphere moves together with the GB. On the high-velocity side, the GB breaks away from the atmosphere and the drag force drops. Eventually, the GB forms a new, much lighter segregation atmosphere that poses less resistance to its motion. MD simulations could offer an effective tool for studying the solute drag effect since they provide all atomic-level details of the GB motion with continuous tracking of the GB velocity and the driving force. Unfortunately, due to the limited timescale of MD simulations and exceedingly low vacancy concentration, simulations of lattice diffusion by the vacancy mechanism are beyond the present-day MD capabilities. Due to this timescale limitation, the common perception has always been that the MD method is not suitable for direct simulations of the solute drag effect.

Contrary to this expectation, it has recently been demonstrated [50] that conventional MD simulations are in fact capable of reproducing the entire force–velocity law predicted by the classical Cahn–Lücke–Stuwe model [29,48,49], including the maximum of the drag force and the low-velocity regime on its left (Figure 1b). In spite of the virtually frozen lattice diffusion, the accelerated (“short-circuit”) GB diffusivity provides enough atomic mobility to ensure that the segregated solute atoms follow the moving boundary. Since GB diffusion is many orders of magnitude faster than lattice diffusion [3,51,52], a moving GB effectively activates solute diffusion in lattice regions swept during its motion.

It should be emphasized that the diffusion process considered in the Cahn–Lücke–Stuwe model [48,49] describes the transport of the solute atoms *across* the moving boundary. The model was developed for a perfectly planar GB moving in the normal direction. As such, this model is essentially one-dimensional and ignores the solute diffusion parallel to the GB plane.

Meanwhile, recent evidence suggests that the in-plane GB diffusion plays a significant role in the solute drag process. It has been demonstrated [50] that a moving GB alters the short-range order in the lattice swept during its motion. The difference between the states of order of the solid solution ahead of and behind the moving boundary creates an additional thermodynamic force that may reduce or amplify the drag effect. Perhaps more importantly, lateral GB diffusion can redistribute the solute atoms in a nonuniform manner, creating concentration fluctuations that look similar to an array of clusters. Such clusters were found in the lattice behind the moving boundary, providing evidence for their formation inside the GB region by GB diffusion-controlled redistribution of the solute atoms. The solute clustering by the moving GB amplifies the resistance to the boundary migration, with the clusters playing a similar role to the disperse particles in the Zener pinning model.

The results mentioned above motivate further research into the role of in-plane GB diffusion in the solute drag process. The goal of the present work was to conduct a deeper and more systematic study in this direction. While the previous work [50] was performed on planar GBs driven by an applied shear stress, here we focus on curved GBs driven by a capillary force. Curvature-driven GB migration is more relevant to the grain growth in nanocrystalline materials. Furthermore, the grains used in the simulations have a nanometer-scale size comparable to those in some nanomaterials. It should also be stressed that the motion of curved GBs is fundamentally different from the motion of planar GBs. In the latter case, the moving boundary carries about the same number of atoms, which are typically organized into structural units whose number remains approximately constant. By contrast, the curved GB motion must be accompanied by annihilation of structural units or creation of new structural units, depending on whether the boundary moves towards or away from the center of curvature [8]. Detailed atomic mechanisms of the creation and annihilation of structural units during the curved GB motion have been reported in the literature [8]. The creation and annihilation of structural units are processes that differ significantly from the translation of existing structural units along with the moving boundary. In other words, the present study probes a different type of GB migration mechanism than in the previous work [50]. There are also significant differences in the solute drag process. A planar boundary can reach a steady state in which it carries a segregation atmosphere containing a fixed amount of the solute. By contrast, the area of a moving curved boundary constantly changes. As a result, the boundary must absorb an increasing amount of the solute during its motion if its area increases or reject some of its segregation atmosphere if the area shrinks. Again, the translation of a fixed segregation atmosphere and the constant absorption or rejection of the solute are different processes that may occur by different atomic mechanisms.

As a model system, we chose Cu-rich solid solutions of the binary Cu–Ag system. We are very familiar with this system from our previous work [50,53,54,55], which enables us to transfer some of the information and methodology. The system has a simple eutectic phase diagram with limited solid-state solubility of both elements and a strong GB segregation trend. In addition, a reliable interatomic potential is available for this system [56].

The simulations were carried out on a bicrystal with a half-loop GB geometry. Half-loop bicrystals were studied extensively in previous atomistic simulations [14,57,58,59,60], although in a different context. An advantage of the half-loop GB geometry is that the GB curvature remains constant as the boundary moves (until it reaches the edge of the sample). This enables us to better control the capillary driving force for GB migration. To gain insights into the segregation formation process and the role of GB diffusion, we start all simulations with a uniform random solution. This initial state can be thought of as obtained by mechanical alloying of the material or by rapid quenching from the melt followed by an isothermal anneal at a higher temperature. The simulations cover a set of temperatures and alloy compositions to explore the temperature and compositional trends.

After describing our methodology in Section 2, we present the solute drag simulation results highlighting the role of GB diffusion in the inhomogeneous redistribution of the solute and its impact on the short-range order in the solution (Section 3.2). In Section 3.3, we report on the composition and temperature dependencies of the GB mobility coefficients. In particular, we find that the alloying causes a much greater decrease in the GB mobility compared with the decrease in the capillary force. This finding confirms that the kinetic stabilization of GBs against grain growth is more effective than the thermodynamic stabilization. In Section 4, we summarize the work and formulate conclusions.

## 2. Methodology

The simulations utilized the embedded-atom method (EAM) [61] potential [56] reproducing a wide spectrum of properties of Cu, Ag, and the Cu–Ag phases, including the phase diagram in agreement with experiments. MD simulations were performed using the Large-scale Atomic/Molecular Massively Parallel Simulator (LAMMPS) [62]. MC simulations utilized the parallel grand-canonical Monte Carlo (ParaGrandMC) code developed by V. Yamakov (NASA) [63,64,65].

**Figure 2:**
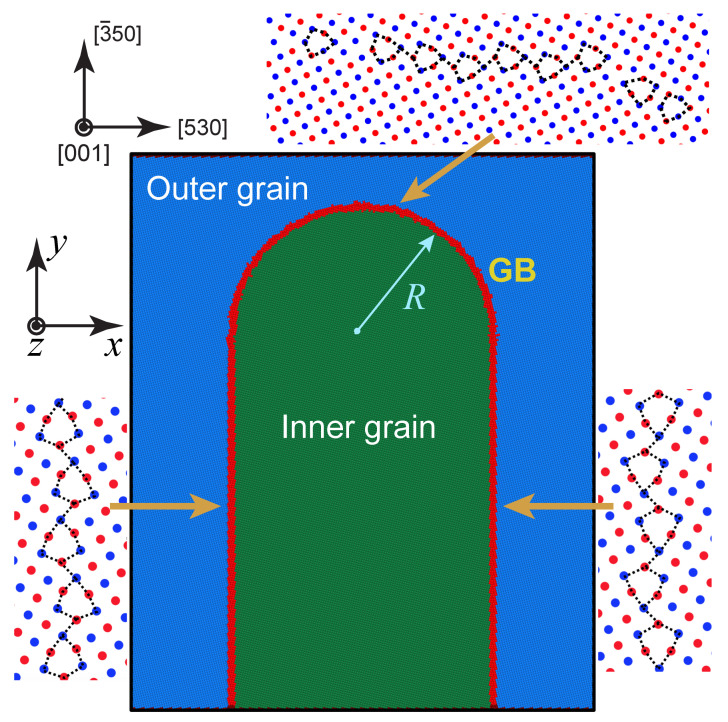
Cu bicrystal with a half-loop GB studied in this work. The GB crystallography is Σ17 [001] with symmetrical tilt inclinations along the vertical portions and at the tip point and asymmetrical tilt inclinations along other curved surfaces. The kite-shaped structural units of the GB structure are outlined. The atoms of the outer and inner grains are shown in blue and green, respectively, with the GB atoms shown in red.

As a model, we chose the Σ17[001] tilt GB with the lattice misorientation angle of $61.93^{{}^{\circ}}$. Here, Σ is the reciprocal density of coincident sites and [001] is the tilt axis. The boundary was created in a bicrystal with the half-loop GB geometry depicted in Figure 2. Approximate dimensions of the simulation block are 52.70×63.34×6.50 nm ($1.83\times 10^{6}$ atoms) with the height and width of the half-loop being 57 nm and 30 nm, respectively. Periodic boundary conditions were applied along *x* and *z* directions with the surface boundary condition in the *y* direction. While the lattice misorientation remains the same along the boundary, the boundary inclination varies. On the straight vertical segments and at the tip of the semicircular segment, the GB is symmetrical with respect to {530} planes. On the curved portions away from the tip, the boundary is asymmetric tilt type.

To obtain the lowest-energy structure of the GB, the system energy was minimized with respect to atomic positions including slight rigid translations of the inner grain. In addition, pairs of atoms separated by less than 0.65 of the first neighboring distance in FCC Cu were replaced by a single atom. On the straight segments, the lowest-energy structure of the boundary is composed of kite-shaped structural units stacked in a zigzag manner (see Figure 2 where the atoms of the alternating (002) planes are shown in red and blue). Rows of these units running parallel to the [001] tilt axis can be viewed as closely spaced intrinsic GB dislocations. This GB structure exactly matches the structure of the Σ17(530)[001] symmetrical tilt GB obtained by previous independent calculations [54,66]. The computed 0 K energy of this boundary, 0.856 J/m${}^{2}$, also matches the results of previous work [54,66], which lends confidence to our GB construction methodology. On the curved portions, the boundary is composed of similar structural units but staggered in a different manner with a varying inter-unit distance accommodating the curved shape of the average GB plane. In preparation for the subsequent simulations, the GB was further relaxed by heating the simulation block to the temperature of 300 K in 0.4 ns and annealing it for another 2 ns in the isothermo-isobaric (NPT) MD ensemble.

To create Cu–Ag alloys, a prescribed amount of Ag was introduced into the sample by random substitution of Cu atoms with Ag atoms. This procedure creates a random solid solution without any short-range order (SRO) or GB segregation. Both SRO and GB segregation could be easily created by Monte Carlo (MC) simulations as was carried out in previous work [54,55]. However, in the present case, we chose to start the GB migration with a completely random solid solution in order to observe the GB effect on the SRO in the lattice regions swept by the GB motion.

To induce GB motion, the sample was heated to the target temperature during a 40 ps MD run and annealed at that temperature in the NPT ensemble. The high-temperature anneal activated GB migration and the inner grain began to shrink. Snapshots of the simulation block were saved at regular time intervals and analyzed using visualization software OVITO [67]. The atoms belonging to each grain were automatically identified in each snapshot using the computer code developed in the previous work [46]. The code finds the lattice orientation in the neighborhood of each atom relative to a reference FCC unit cell. The atoms located in the GB region were identified using the centrosymmetry parameter and the bond-angle analysis [67] and were excluded from the grain orientation analysis. However, after the grain orientations were determined, some of the excluded atoms were assigned to the grains if at least 90% of their neighbors had the same orientation.

Two independent methods were employed to track the GB velocity during the simulation. In the first method, the tip of the half-loop was located by fitting the equation of a semicircle to the semicircular GB portion revealed by non-structural atoms identified by OVITO. Knowing the instantaneous height *h* of the inner grain, the GB velocity *V* was found from its time dependence. The second method required counting the number of atoms *N* located inside the inner grain, which was determined by the lattice orientation analysis mentioned above. The GB velocity was found from the Equation (1).

(1) $$V=\frac{\Omega }{wl}\frac{dN}{dt},$$

where Ω is the atomic volume, *w* is the grain width (distance between the planar segments), and *l* is the grain thickness in the *z*-direction. The derivative was evaluated numerically. Both methods gave the same magnitude of the GB velocity within the statistical scatter of the data, lending additional confidence to our methodology.

To evaluate the driving force for the boundary motion, we needed to know the GB free energy γ as a function of temperature and alloy composition. To obtain a lower bound of the driving force, we computed the equilibrium values of γ by separate calculations for the plane Σ17(530)[001] symmetrical tilt GB. This boundary was created in a periodic simulation block with approximate dimensions of 10.54×21.13×10.48 nm ($1.97\times 10^{5}$ atoms). Ag atoms were introduced into the system by semigrand canonical MC simulations implemented at a chosen temperature *T*. The chemical potential difference between Ag and Cu was fixed at a value that gave the targeted chemical composition in the grains. The trial MC moves included random displacements of randomly selected atoms and a random re-assignment of their chemical species to either Ag or Cu. They additionally included random changes in the dimensions of the simulation block with simultaneous rescaling of the atomic coordinates. The trial moves were accepted or rejected by the Metropolis algorithm. This simulation scheme ensured zero-stress conditions in all three Cartesian directions and produced a thermodynamically equilibrium distribution of the Ag atoms in the GB region and inside the grains. The simulations covered temperatures between 600 and 1100 K and alloy compositions from pure Cu to 2 at.%Ag.

The amount of equilibrium Ag segregation was measured by the excess number of Ag atoms per unit GB area at a fixed total number of atoms:

(2) $$\left[N_{\mathrm{Ag}}\right]=N_{\mathrm{Ag}}-N\frac{N_{\mathrm{Ag}}^{\prime }}{N^{\prime }}.$$

Here, $N_{\mathrm{Ag}}$ and $N_{\mathrm{Ag}}^{\prime }$ are the numbers of Ag atoms per unit area in two regions with and without the GB, respectively. Likewise, *N* and $N^{\prime }$ are the total numbers of Cu and Ag atoms in the respective regions. Both regions were represented by layers parallel to the GB plane. In this work, we measure the alloy composition $c_{\mathrm{Ag}}$ by the atomic percentage of Ag atoms. Given the relatively large dimensions of the system, the average chemical composition of the alloy closely represents the chemical composition $N_{\mathrm{Ag}}^{\prime }/N^{\prime }$ in the interior regions of the grains.

The GB free energy would normally be obtained by thermodynamic integration as in our previous work [54,55]. However, given that the Ag concentration is small, we have simplified the calculations by extracting γ from the dilute solution form of the Gibbs adsorption equation:

(3) $$\gamma \left(c_{\mathrm{Ag}},T\right)=\gamma_{0}\left(T\right)-kT\left[N_{\mathrm{Ag}}\right],$$

where $\gamma_{0}\left(T\right)$ is the GB free energy in pure Cu and *k* is Boltzmann’s constant. The accuracy of this approximation will be assessed below.

## 3. Results and Discussion

### 3.1. Grain Migration in Pure Cu

**Figure 3:**
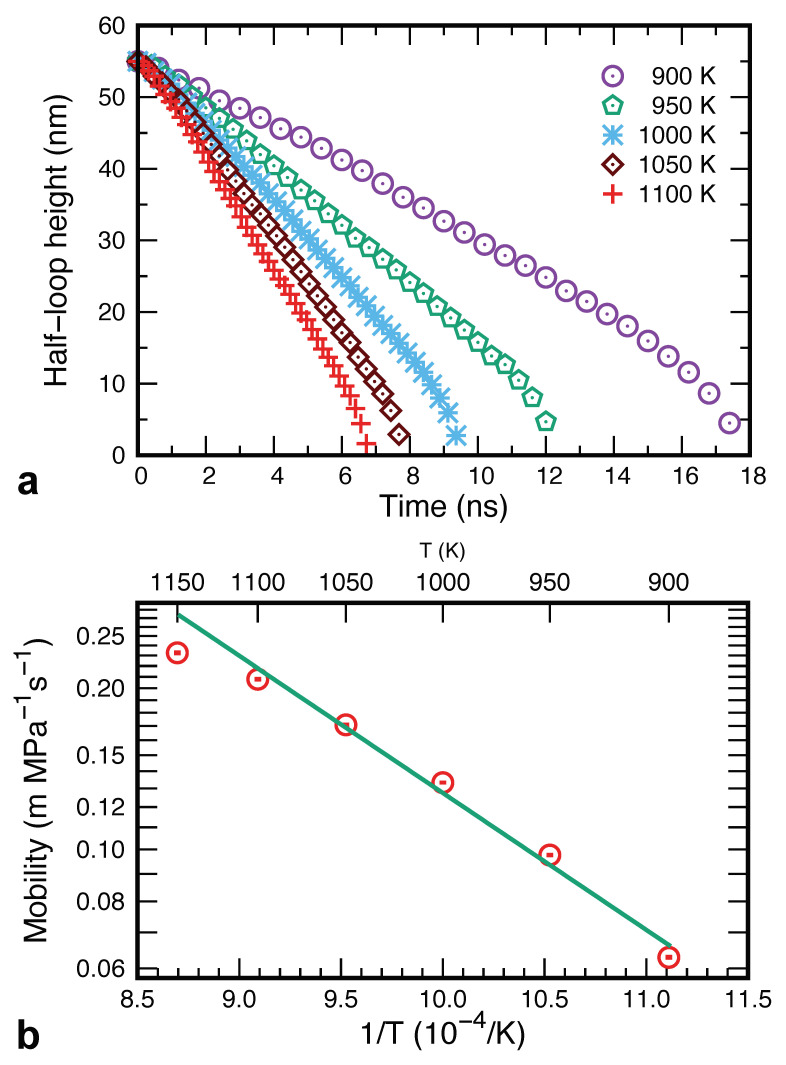
GB dynamics in MD simulations of pure Cu. (**a**) The grain height as a function of time at several temperatures. (**b**) Arrhenius diagram of GB mobility. The points in (**b**) correspond to the temperatures indicated in (**a**), with the straight line representing the linear fit to determine the effective activation energy as GB migration.

To provide a baseline for comparison with the Cu–Ag system, we first performed simulations of the pure Cu system. As expected, the inner grain was found to shrink and eventually disappear at all temperatures tested. Figure 3 demonstrates the decrease in the grain height *h* as a function of time at several temperatures. The data points represent individual snapshots generated during the MD simulations. Since the straight GB segments do not move, the curvature of the moving part remains constant. The plots show that the GB motion follows a linear law for most of the simulation time, allowing us to extract the constant GB velocity at each temperature. At the end of the simulation, the grain shrinkage accelerates, becoming nearly parabolic. This happens when the semicircular GB portion reaches the bottom of the simulation block. The subsequent grain shrinkage is accompanied by increasing GB curvature, leading to the parabolic kinetics observed previously during the shrinkage of spherical and circular grains [8,45,68].

The capillary force driving the GB migration is

(4) $$P=\frac{\gamma }{R}$$

In the linear regime, the curvature radius is R=w/2=const. Since the velocity also remains constant, we can assume that the boundary follows the linear dynamics V=MP with a constant mobility coefficient *M*. The latter can be extracted from the simulation data provided that we know the GB free energy. Fortunately, the temperature dependence of γ for this particular boundary in pure Cu was previously calculated by the thermodynamic integration method [54] and was used in this work. An Arrhenius plot of the GB mobility obtained is shown in Figure 3. The mobilities have a reasonable order of magnitude comparable to that in previous simulations of Cu GBs using the same interatomic potential [69,70,71].

Generally, the mobility coefficients need not follow the Arrhenius law since the dominant GB migration mechanism may change with temperature. For example, low temperatures could be dominated by collective atomic mechanisms as suggested by recent MD simulations [72]. It is, therefore, unsurprising that the data points in Figure 3 display a slight downward curvature. Nevertheless, we found it useful to estimate an effective activation energy of GB migration, $E_{m}$, by a linear fit to all points. This gives the activation energy of $E_{m}=0.47\pm 0.03$ eV, where the error bar indicates one standard deviation. To put this number into perspective, this energy is lower than the activation energy of Cu self-diffusion in the perfect FCC lattice (1.99 to 2.03 eV [73,74]) but is comparable to typical values of the activation energy of GB self-diffusion. For example, the experimental activation energy $E_{d}$ of GB self-diffusion in polycrystalline Cu is $E_{d}=0.75\pm 0.015$ eV [74]. It is known, however, that the GB diffusivity strongly depends on the GB bicrystallography [51,75,76,77]. Previous atomistic calculations have shown that $E_{d}$ tends to decrease with increasing GB energy γ [78]. For γ values close to the energy of the present GB, the predicted $E_{d}$ values are scattered over the range from 0.5 to 0.8 eV [78]. More recent calculations for two different structures (phases) of a Σ5 [001] tilt GB gave $E_{d}=0.5$ eV and 0.7 eV, respectively [77]. Given the uncertainties associated with this comparison (different GBs and simulation/measurement methods), we can conclude that the GB migration energy obtained here reasonably correlates with the activation energy of GB self-diffusion in Cu.

### 3.2. Grain Boundary Migration in the Random Alloy

We next present the results for GB dynamics in the Cu–Ag alloys. As expected, the random distribution of Ag atoms in Cu produces a friction effect reducing the rate of GB migration in comparison with the pure Cu case. As the Ag concentration $c_{\mathrm{Ag}}$ increases, the GB moves slower, until at about 1.5 at.%Ag it stops moving on the MD timescale.

**Figure 4:**
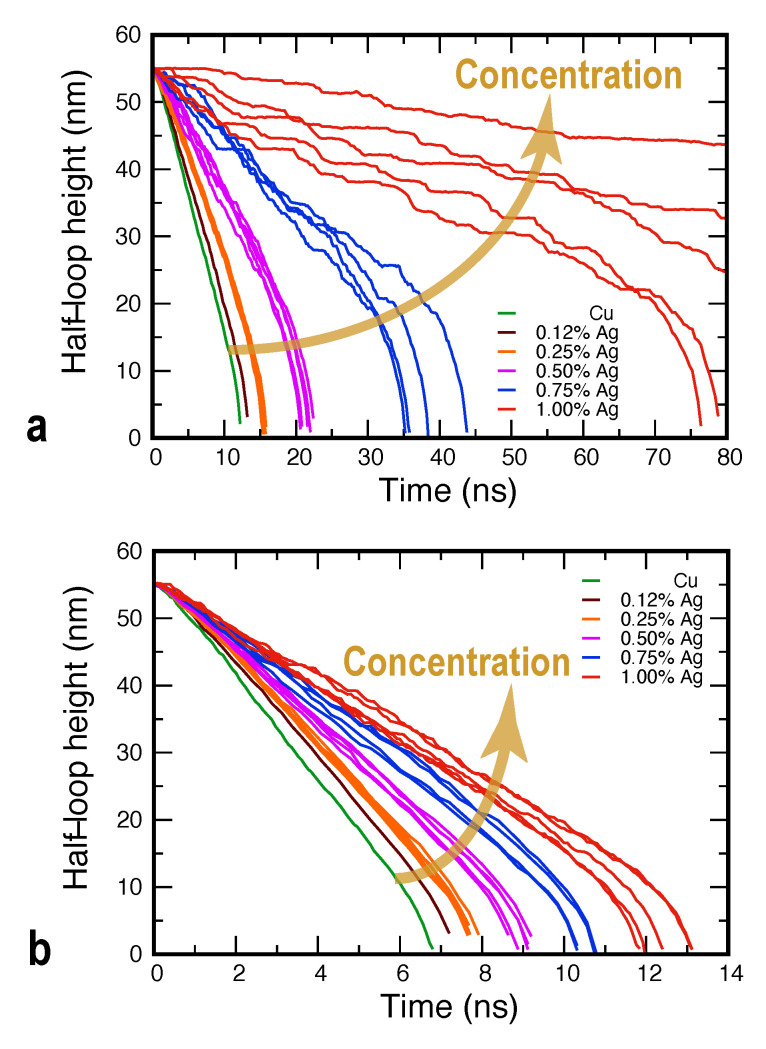
GB dynamics in MD simulations of Cu–Ag alloys at the temperatures of (**a**) 950 K and (**b**) 1100 K.

Figure 4 shows representative GB displacement–time curves for different alloy compositions at two different temperatures. The plot illustrates the general trend that the slope of the curves (and thus the GB velocity) is smaller than in pure Cu and decreases with increasing $c_{\mathrm{Ag}}$. To demonstrate the statistical nature of the GB migration process, at some of the temperatures, several curves were computed for the same chemical composition. Such curves were obtained using different random seeds when introducing the Ag atoms into the alloy, which resulted in statistically independent trajectories during the subsequent MD simulations. At high temperatures and low Ag concentrations, such sets of curves form tight bunches (Figure 4b), indicating that the GB dynamics are well reproduced. At lower temperatures and/or higher solute concentrations, the results display a greater variability, as exemplified by the curves for the 1 at.%Ag alloy at 950 K (Figure 4a). Furthermore, the increase in the statistical scatter is accompanied by a dynamic transition from nearly continuous GB motion to a stop-and-go motion manifested in the stepwise shape of the GB displacement–time curves. As will be discussed later, the increased statistical variability and the stop-and-go GB motion are caused by the formation of a random distribution of solute clusters driven by GB diffusion.

Examination of the GB positions, shapes, and the solute atom distributions suggests that the stop-and-go behavior is a manifestation of dynamic instability of the GB motion caused by its interaction with the solute atoms. Specifically, the attraction of Ag atoms to the GB causes the solute drag effect, resulting in alternating periods of pinning and unpinning similar to the stick–slip behavior in sliding friction [79]. It is important to emphasize that the pinning–unpinning process observed here is not due to the GB interaction with individual solute atoms. It is caused by the formation of small groups of Ag atoms (clusters) inside the moving GB, which strongly resist its motion by a Zener pinning type mechanism. The boundary eventually breaks away from the clusters and continues to move towards the center of curvature, only to be pinned again by new clusters. The alternating pinning (arrest) and break-away periods produce the staircase pattern clearly seen in the displacement–time curves (Figure 4a). The fact that it takes time for the boundary be stopped after it breaks away from the pinning points reflects the cumulative nature of the solute drag process. This time is required for the diffusion-controlled formation of new clusters strong enough to counterbalance the capillary force. A critical amount of clusters can even pin the boundary in place, which is observed at a high enough concentration of the solute.

**Figure 5:**
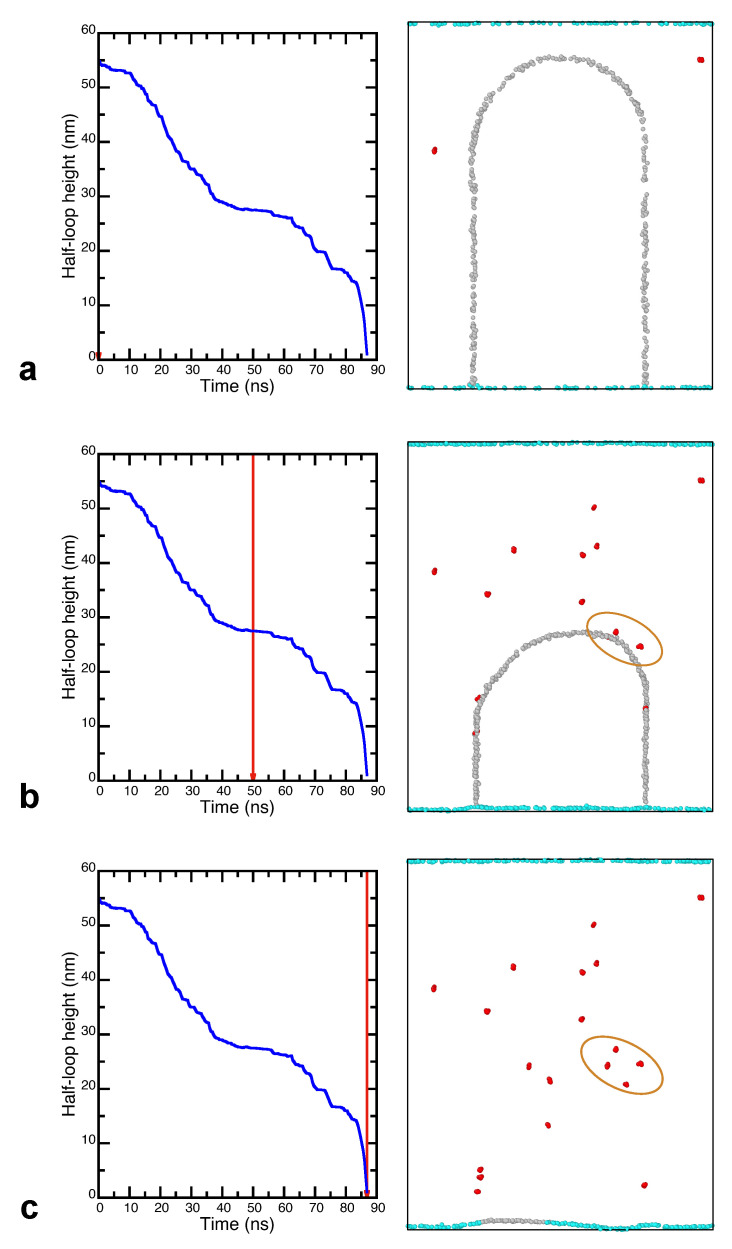
Displacement–time curve for a GB moving in the 1.25 at.%Ag alloy at the temperature of 1000 K (left column) in comparison with GB images at the moments of time indicated by the vertical red line. (**a**) Initial GB. (**b**) GB after 50 ns of simulation. (**c**) GB at the end of the simulation. Atoms with FCC environment are invisible. The GB atoms are shown in gray and the Ag clusters containing five or more atoms are shown in red. The oval indicates the group of clusters responsible for the plateau in the displacement curve. Note how, in (**b**), the GB bows out toward these clusters.

**Figure 6:**
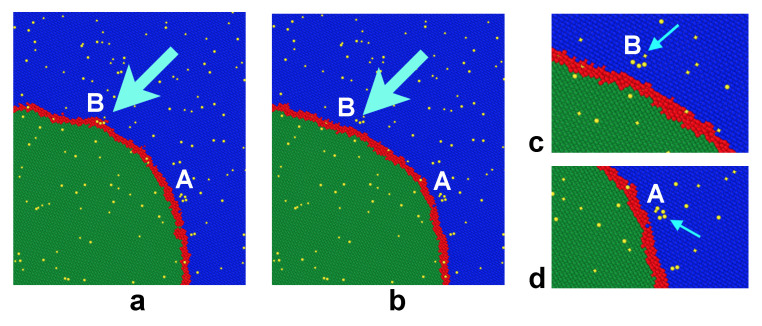
Interaction of the moving GB with solute clusters. Cu atoms of the outer and inner grains are shown in blue and green, respectively, with GB atoms shown in red and Ag atoms in yellow. The GB is moving towards the lower left corner of the images. In (**a**), the GB has just separated from the group of atoms (cluster) A but is still pinned by the cluster B. In (**b**), the GB broke away from the cluster B. Panels (**c**,**d**) are zoomed in views of the two clusters. The cyan arrows point to the clusters.

The process described above is illustrated in Figure 5 for the GB moving in the 1.25 at.%Ag alloy at 1000 K. In this case, the displacement–time curve displays a prominent plateau between 40 and 60 ns in addition to many smaller plateaus before and after this time period. Ag clusters were identified in the structure as groups of Ag atoms connected with each other by nearest neighbor bonds. Only clusters containing five or more atoms are shown in the images, although some of them contain up to 10 atoms. Side-by-side comparison of the displacement curve with the snapshots taken between 40 and 60 ns shows that the stagnation period was primarily caused by a group four clusters formed one after another on the right-hand side of the half-loop. These clusters are highlighted in the images and are illustrated separately in more detail in Figure 6. Once the GB breaks away from the pinning clusters and starts moving, it exerts a capillary force on neighboring GB regions and helps them detach from their pinning points. Thus, the unpinning process quickly propagates along the curved part of the GB and produces its precipitous displacement toward the center of curvature. This behavior is similar to the “unzipping” mechanism described previously [45] and explains the existence of the nearly vertical segments of the displacement–time curves between the horizontal segments caused by the GB arrest.

We emphasize that the Ag clusters shown in Figure 5 and Figure 6 did not exist in the initial random alloy. They simply could not form because the lattice diffusion is virtually frozen on the timescale of the present simulations. A few clusters could occasionally pre-exist in the initial solution by pure chance. Two of such clusters can be seen in the outer grain in Figure 5a. However, the massive formation of new clusters behind the moving boundary illustrated in Figure 5c required extensive diffusion of the Ag atoms. The enhanced diffusive mobility of the solute atoms was provided by the short-circuit diffusion along the moving GB.

The formation of Ag clusters in the lattice traversed by the moving boundary does not mean that a second, Ag-rich phase precipitates from the solution. The grains remain a single-phase solid solution. The cluster formation is only a manifestation of the SRO that did not exist in the initial state and was created in the wake of the moving boundary due to GB diffusion. On a longer timescale, such clusters would spontaneously form and dissolve at random locations due to thermal fluctuations. However, on the MD timescale, the clusters left in the lattice behind the moving GB remain virtually “frozen” in their instantaneous configurations due to the slowness of the lattice diffusion.

**Figure 7:**
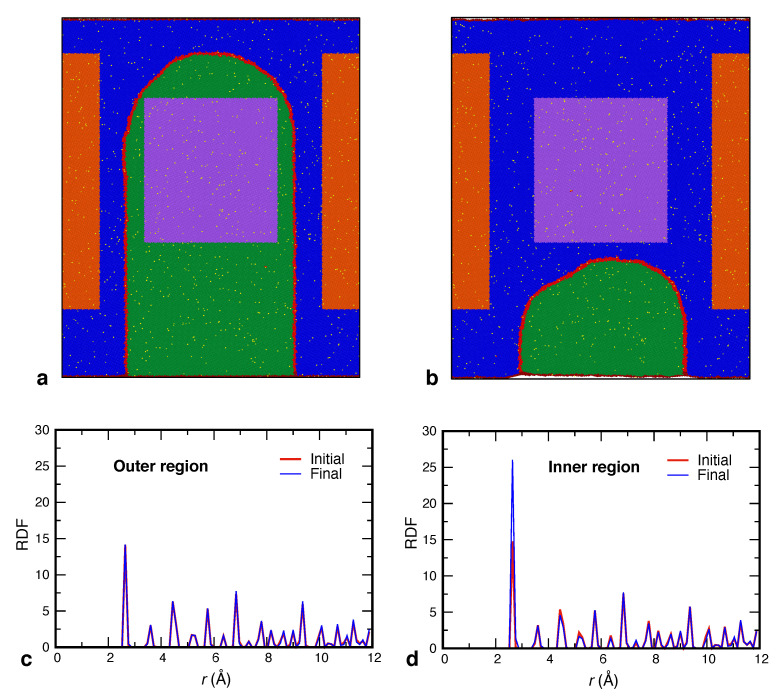
(**a**,**b**) GB position in the beginning of the MD simulation (**a**) and closer to the end (**b**). Cu atoms of the outer and inner grains are shown in blue and green, respectively. The GB atoms are shown in red and Ag atoms in yellow. The purple rectangular region is selected to show the SRO formation and is swept by the GB motion. The orange region lies completely in the outer grain and is unaffected by the GB motion. The graphs below compare the Ag–Ag RDFs in the two states shown (**a**,**b**) for the outer (**c**) and inner (**d**) regions, respectively. Note the significant rise in the first peak in the region traversed by the moving boundary.

**Figure 8:**
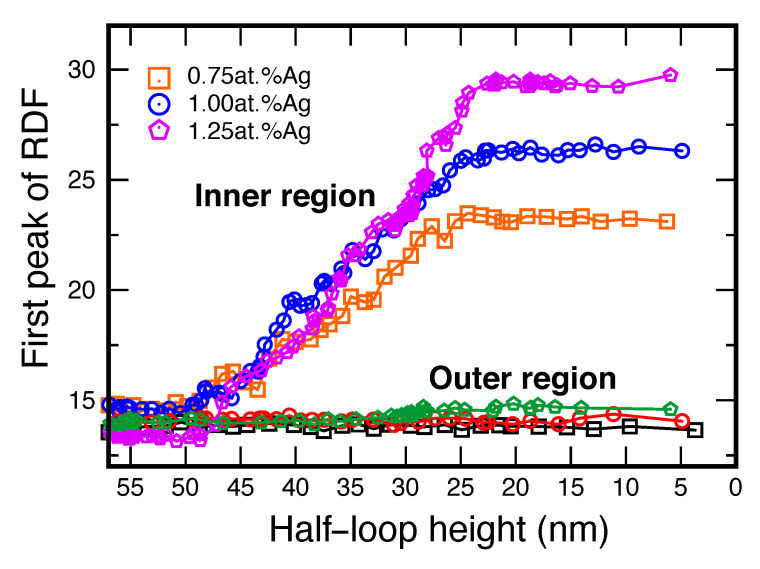
Change in the RDF’s first peak height (measure of the SRO) in the inner and outer rectangular regions shown in Figure 7 during the shrinkage of the inner grain. The results are shown for three alloy compositions indicated in the key at the temperature of 1000 K. Note that the SRO in the outer region changes very little, while the SRO in the inner region increases as the GB sweeps through its volume and levels out after the GB exits the region.

To further demonstrate this effect, we directly examined the SRO in the lattice regions swept by the boundary. As a quantitative measure of SRO, we chose the height of the first peak of the radial distribution function (RDF) of Ag atoms. The simulation setup is illustrated in Figure 7. A rectangular region shown in purple was selected inside the inner grain. The thickness of the region normal to the page coincides with the thickness of the simulation block. This region is swept by the moving GB as the inner grain shrinks as shown in Figure 7a,b. A similar rectangular region inside the outer grain (shown in orange) was chosen as a control system. In the initial state, the RDFs computed in both regions are nearly identical (Figure 7c,d) and coincide with the RDF of Cu–Cu pairs (not shown) as expected for a purely random solution. After the GB passes through the inner region, the height of the first RDF peak nearly doubles, reflecting the formation of a significant SRO. By contrast, the RDF in the outer region does not show any significant changes. Figure 8 shows that the SRO forms gradually as the boundary sweeps through the inner region and stops changing once the GB exits the region while the SRO in the outer region remains unchanged. Since SRO formation requires rearrangement of the solute atoms, this behavior provides direct evidence for the acceleration of atomic diffusion in the lattice caused by the GB motion.

### 3.3. The Alloying Effect on GB Mobility

**Figure 9:**
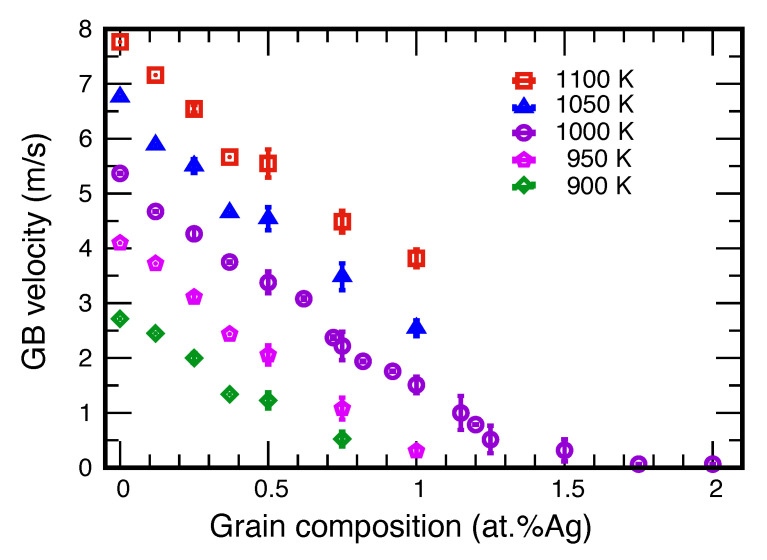
GB velocity as a function of temperature and alloy composition. The two zero-velocity points at 1000 K represent the GB arrest at concentrations above 1.5 at.%Ag.

Figure 9 depicts the GB velocity as a function of temperature and chemical composition, showing the expected trend of accelerating with increasing temperature (thermal activation) and decreasing solute concentration (less solute drag). As mentioned above, the observation of constant GB velocity under the imposed constant GB curvature 1/R suggests that, in the present case, the GB migration follows the linear constitutive relation V=MP with the capillary driving force *P* given by Equation (4). Since the GB free energy γ is usually unknown and not easy to compute, most studies interpret the results in terms of the so-called reduced GB mobility Mγ. In this work, we set the goal of extracting the GB mobility *M* itself. This requires the knowledge of the absolute values of γ.

For elemental materials, the calculation of γ is a tedious but straightforward procedure [54,80,81,82,83]. For an alloy system, the situation is more complex, especially in the presence of GB motion. A moving GB is generally not in thermodynamic equilibrium with the grains, making the very definition of the GB free energy fundamentally ambiguous [84]. In this work, we chose to estimate γ by computing its upper and lower bounds. The values of γ for pure Cu give an upper bound since solute segregation to the GB can only reduce its free energy. The values of γ computed under the assumption of thermodynamic equilibrium between the GB and the grains give a lower bound since this situation maximizes the solute-induced free energy reduction.

**Figure 10:**
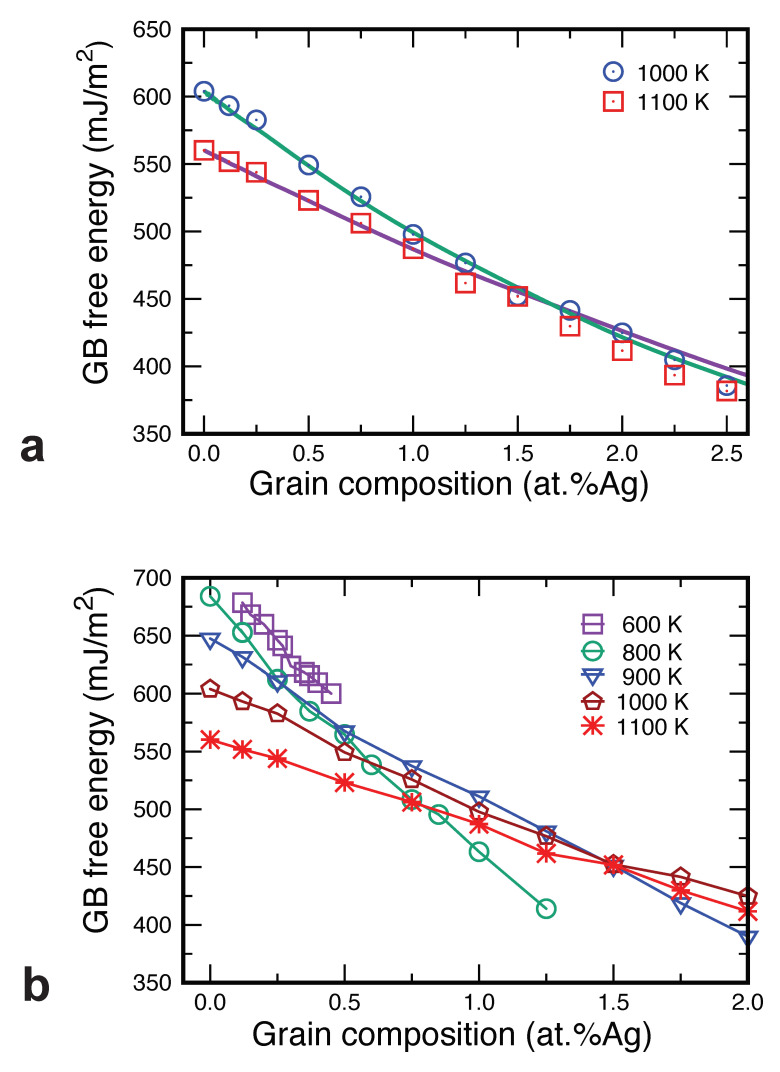
Composition and temperature dependencies of the equilibrium GB free energy in the Cu–Ag solid solution. (**a**) Comparison of the exact free energy obtained by thermodynamic integration [54] (lines) with the dilute solution approximation by Equation (3) (points). (**b**) Calculations from Equation (3) for several temperatures and alloy compositions.

For pure Cu, we utilized the values of γ computed previously [54]. For the Cu–Ag alloys, we could have obtained the equilibrium values of γ by the thermodynamic integration method applied in previous work [54,83]. Instead, given the small Ag concentrations studied here, we chose to approximate $\gamma (c_{\mathrm{Ag}},T)$ by the dilute solution form of the adsorption equation given by Equation (3). The solute segregation $\left[N_{\mathrm{Ag}}\right]$ entered into this equation was computed from Equation (2) [55]. To validate this approximation, in Figure 10a, we compare the predictions of Equation (3) with exact calculations by thermodynamic integration at two temperatures [54]. The agreement between the two methods is very reasonable at both temperatures, especially for alloy compositions below 1.5 at.%Ag, which is the composition range in which the GB velocity could be reliably measured. This agreement justifies the application of Equation (3) to other temperatures, with the results displayed in Figure 10b. Note that the low-temperature curves cover a narrower concentration range due to the lower solid solubility limit of Ag in Cu at these temperatures.

We acknowledge that the calculations of both the upper and lower bounds of γ rely on the assumption that the planar GB adequately represents the free energy of the curved GB which is actually moving in the present simulations and whose local inclination varies along the semicircular portion. This is a significant approximation, but it does not affect our final conclusions about the relative magnitude of the thermodynamic and kinetic factors of GB migration. As discussed below, the variations in the GB free energy are small in comparison with the variations in the GB mobility.

**Figure 11:**
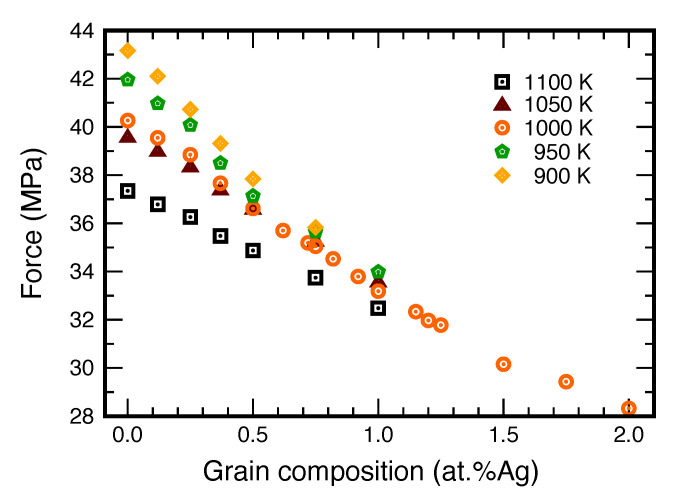
The capillary driving force for GB migration as a function of temperature and alloy composition.

Knowing the GB free energies, we can now estimate upper and lower bounds of the capillary driving force P=γ/R (Figure 11). The difference between the two bounds is the change in *P* between pure Cu and the Ag concentration of 1.5 at.%. It can be seen that the maximum segregation-induced reduction in the driving force varies between approximately 10% and 20%, depending on the temperature.

**Figure 12:**
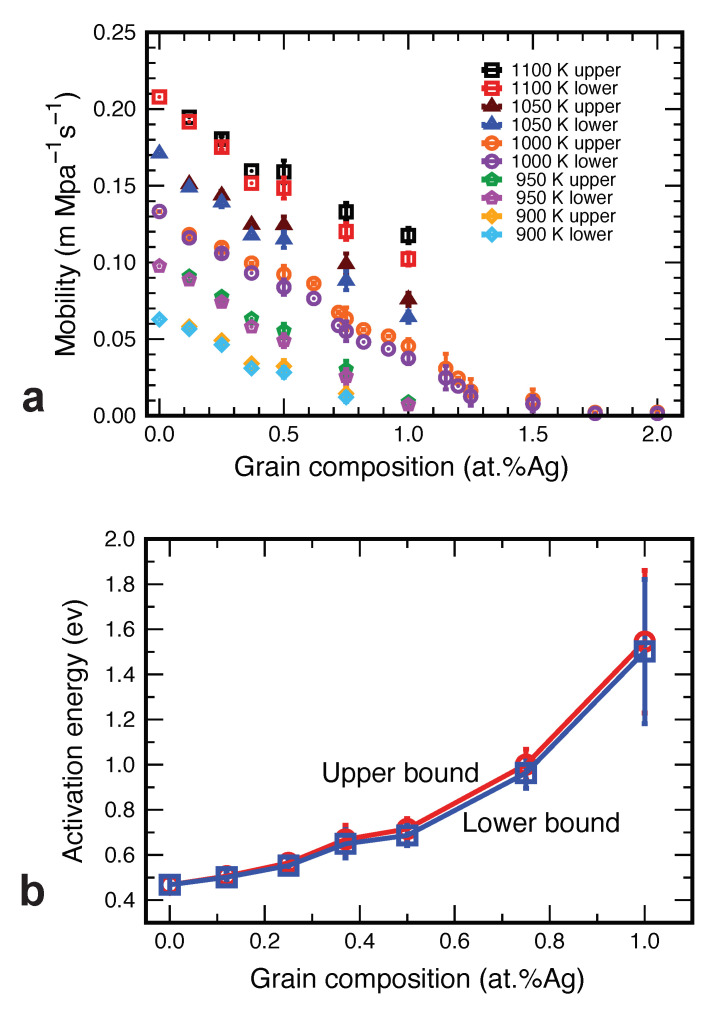
(**a**) The upper and lower bounds of the GB mobility coefficient as a function of temperature and alloy composition. (**b**) The upper and lower bounds of the activation energy of GB mobility as a function of alloy composition.

The computed GB mobility coefficients M=V/P are plotted in Figure 12a. Two features of the plot are notable. First, the gap between the upper and lower estimates of *M* is narrow relative to the changes in the magnitude of *M* between the temperatures tested. This makes the obtained mobility coefficients well-defined GB properties despite the uncertainty in the GB free energy. Second, the mobility exhibits much greater variations with temperature and chemical composition than does the GB free energy. For example, the mobility drops a factor of four to ten between the temperatures of 1100 K and 900 K. With the addition of 1.5 at.%Ag, the mobility drops another factor of two to ten, depending on the temperature. In other words, the GB segregation has a much greater effect on the GB mobility than on the capillary driving force (10% and 20%).

Finally, replotting the mobility data in Arrhenius coordinates, we estimated the activation energy of the GB mobility as a function of alloy composition (Figure 12b). The fact that the activation energy increases with the solute concentration is not surprising as it reflects the solute drag effect. However, the magnitude of the effect is remarkable. The addition of only 1 at.% of Ag atoms increases the activation energy of GB migration by a factor of three.

## 4. Conclusions

We have studied the solute drag on GBs in Cu–Ag solid solutions by MD simulations. The simulations cover a relatively wide single-phase domain on the Cu-rich side of the Cu–Ag phase diagram. By starting the simulations from a purely random solution, we were able to track the effect of GB motion on the state of short-range disorder in the solution. Although the lattice diffusion is virtually frozen under the simulation conditions, the GB significantly accelerates diffusion in the lattice regions swept by its motion. This acceleration is manifested in the formation of the initially absent SRO in the solution behind the moving boundary. The SRO jump across the boundary causes an additional driving force for GB migration. More importantly, the accelerated diffusion parallel to the GB plane produces a nonuniform redistribution of the solute atoms in the boundary, which can strongly affect the solute drag. The compositional fluctuations in the segregation atmosphere are akin to solute clusters that impose an additional drag force for the GB motion by a mechanism similar to the Zener pinning.

The results reported here point to an important role of lateral GB diffusion in the solute drag effect, which is not included in the classical models [29,48,49] and overlooked in all subsequent studies. Further investigations of the role of the lateral GB diffusion deserve research efforts in the future.

To our knowledge, this is the first atomistic study reporting on absolute values of GB mobility coefficients in alloy systems. A comparison of the values obtained with previous studies is problematic even for pure Cu. Most of the previous work was done for planar GBs driven by an applied shear stress [45,50], the elastic energy differential across the boundary [70,71], or the synthetic driving force [69,85,86,87]. Those cases are different from the curvature-driven migration process studied here, in which the GB must constantly create or eliminate structural units. In an alloy system, the GB must also constantly absorb or reject some of the segregated solute. Proper comparison will become possible in the future as more simulation studies are performed for capillary-driven GB migration in different alloy systems. In particular, it will be interesting to explore the effect of the type of solute–solvent chemical interactions of the solute drag. For example, in contrast to the Cu–Ag system with a miscibility gap on the phase diagram, systems with intermetallic phases on the phase diagram may show a different solute drag behavior. Interstitial solutes present another interesting case to study.

It was found that even alloying on the 1 at.% level significantly increases the activation energy of GB migration and reduces the mobility coefficient by more than an order of magnitude, whereas the GB free energy only decreases by 10 to 20%. This observation suggests that the kinetic approach to grain stabilization in nanomaterials is more effective than the thermodynamic stabilization aiming to reduce the GB free energy.

## Data Availability

The compuational data presented in this study are available from the authors upon reasonable request.
